# Hidden Problem of COVID-19: Behavioral Surveillance and Its Preventive Measures Among Rural Adult Population in Tamil Nadu, India

**DOI:** 10.7759/cureus.41272

**Published:** 2023-07-02

**Authors:** Sundaram Ramkumar, Srinivasan Vijayalakshmi, Shagirunisha Rizvana, Ponmozhi Thirumoolan, Pavatharani Paulraj, Pavithra Thirunavukkarasu, Oveya Senthilkumar

**Affiliations:** 1 Community Medicine, Dhanalakshmi Srinivasan Medical College and Hospital, Perambalur, IND

**Keywords:** rural area, behavioural surveillance, knowledge, preventive measures, covid-19

## Abstract

Background

COVID-19-appropriate behavior has been recommended by the Government of India to reduce the spread of the disease. However, a lack of awareness, inadequate knowledge, or improper practices regarding personal protective measures have contributed to the ongoing cases in India. Therefore, this study aimed to assess the pattern of behavioral surveillance related to COVID-19 and its preventive measures and also to study the impact of knowledge of COVID-19 on their behavior among the rural population in Tamil Nadu, India.

Methods

A descriptive cross-sectional study was conducted among the rural adult population in Siruvachur Village, Perambalur district, Tamil Nadu, from June to December 2022. The study included adult participants aged 18 years and older who had no previous history of COVID-19 disease. The participants were selected using a systematic random sampling method. A pre-designed and semi-structured questionnaire was used to assess their awareness of COVID-19, knowledge of symptoms, preventive measures, and actual behavioral practices. The collected data were analyzed using IBM Corp. Released 2011. IBM SPSS Statistics for Windows, Version 20.0. Armonk, NY: IBM Corp. The chi-square test and linear regression were employed to assess the association and strength between behavior and knowledge of COVID-19 preventive measures. A p-value of less than 0.05 was considered statistically significant.

Results

According to the results, 94.7% of the participants reported being aware of COVID-19, with fever being recognized as the most common symptom. Regarding specific preventive measures, 71.5% of participants demonstrated adequate knowledge of handwashing, while only 55.3% had sufficient knowledge of social distancing norms. In terms of mask usage, 62% reported having adequate knowledge of how to use masks to prevent COVID-19 transmission. However, despite having adequate knowledge, the study found that only 11.3% of participants consistently used masks in outdoor settings. This indicates a significant gap between knowledge and actual behavior in mask usage. Furthermore, a significant association was found between knowledge and behavioral practices related to COVID-19 preventive measures, such as mask usage and social distancing. In other words, participants who possessed greater knowledge of these measures were more likely to exhibit corresponding behaviors. On the other hand, the study did not find a significant impact of handwashing knowledge on actual handwashing behavior (p>0.05).

Conclusion

Despite a relatively high level of knowledge and awareness, there is a significant gap between knowledge and actual behavioral practices, particularly in terms of mask usage and hand hygiene. These findings highlight the need for targeted interventions to bridge the gap between knowledge and behavior in COVID-19 preventive measures, particularly in terms of consistent mask usage and adherence to social distancing norms. Efforts should focus not only on increasing knowledge but also on promoting behavior change through effective education, awareness campaigns, and practical demonstrations of proper preventive measures.

## Introduction

Coronavirus disease 2019 (COVID-19) is a global pandemic caused by the severe acute respiratory syndrome coronavirus 2 (SARS-CoV-2) [1]. It is a highly infectious disease characterized by mild to severe symptoms such as fever, cough, myalgia, dyspnea, and respiratory failure. Recognizing its virulence and rapid spread, the World Health Organization (WHO) declared the COVID-19 outbreak a Public Health Emergency of International Concern on January 30, 2020 [1].

In India, there have been 44,991,393 confirmed cases and 531,896 COVID-19-related deaths reported as of June 5, 2023 [2]. As per WHO, the prevalence of COVID-19 in India is significant, with 3,260 confirmed cases per 1 lakh population and 38.54 deaths per 1 lakh population [2]. The unpredictable nature of COVID-19 and the emergence of new variants have threatened the healthcare system and routine management, leading to an ongoing battle with the COVID-19 pandemic. Limited knowledge of the epidemiological evidence of the disease and the ongoing vaccine campaign has presented serious challenges to the Government of India [3].

To mitigate the spread of COVID-19 and prolong the time until its control, the general population needs to engage in preventive measures. Despite the ongoing pandemic, educating, involving, and mobilizing the general population as active participants can enhance the population's health emergency preparedness and reduce overall vulnerability [4]. Recent studies have highlighted that individual behaviors, including practicing personal hygiene and maintaining social distancing, can significantly decrease the morbidity and mortality rates of COVID-19 [5,6].

Although COVID-19-appropriate behaviors have been recommended by the Government of India, the lack of awareness, inadequate knowledge, and improper practices regarding personal protective measures have led to ongoing cases (44,993,543 confirmed cases of COVID-19) in India [2]. Therefore, this study aimed to assess the pattern of behavioral surveillance of COVID-19 and its preventive measures among the rural population in Tamil Nadu and also aimed to assess the influence of knowledge on the adoption of COVID-19-appropriate behavior among the study subjects.

## Materials and methods

Study design

After obtaining permission from the Institutional Ethics Committee (Human Study), a descriptive cross-sectional study was conducted among the rural adult population in Siruvachur village, Perambalur district, Tamil Nadu, India, from June to December 2022.

Study area

The present study was conducted in Siruvachur village, which falls under the administrative jurisdiction of Perambalur. Perambalur is located in the southern part of Tamil Nadu and has a population of 4,500.

Study population

Studying populations aged more than 18 years is crucial for understanding the impact of COVID-19 preventive measures, as this age group often plays a significant role in disease transmission and adherence to public health guidelines. So, the study population of 18-year-olds and older who were willing to participate were included in the study. Individuals with a history of COVID-19 were excluded from the study.

Sample size calculation

The sample size for the study was determined based on the prevalence of positive practice on COVID-19 preventive measures from a previous study [7]. To determine the sample size for a finite population of 4,500 with a prevalence of 64% and a margin of error of 5%, the following calculation was performed:

n = (Z^2 * p * (1 - p) * N) / [(Z^2 * p * (1 - p)) + (E^2 * (N - 1))]

Given:

Population size (N) = 4,500

Prevalence (p) = 0.64 (64%)

Confidence level (Z) = For a 95% confidence level, Z is approximately 1.96 (standard value)

Margin of error (E) = 0.05 (5%)

Substitute these values into the formula:

n = (1.96^2 * 0.60 * (1 - 0.64) * 4500) / [(1.96^2 * 0.64 * (1 - 0.64)) + (0.05^2 * (4500 - 1))]

n = 379. Considering a 10% non-response rate, the estimated sample size was approximately 422.

Sampling technique 

The study included the adult population residing within the jurisdiction of the Rural Health Training Centre, which is affiliated with a tertiary hospital located in Siruvachur village. The total population of adults in this area was 4,500. To ensure a representative sample, a systematic random sampling method was utilized. The sampling interval was determined to be 10.6, and for ease of data collection, every 10th house was selected for inclusion in the study. House-to-house data collection was done, and the houses that were locked or where adults were not available during data collection were not included, and the next house was selected instead. One elderly adult from each selected house was included as a participant, resulting in a total of 422 study participants. 

Study tool

A pre-validated semi-structured questionnaire was employed to collect data on socio-demographic profiles, knowledge, and behavior related to COVID-19 preventive measures. The calculated Cronbach's alpha value of the questionnaire was 0.739, indicating a satisfactory level of internal consistency. This suggests that the items within the questionnaire consistently measure the same underlying construct.

The questionnaire encompassed socio-demographic aspects, including age, gender, education, employment status, employment-related frequent contacts with COVID-19, and socio-economic status (as per the Modified BG Prasad Classification).

The knowledge component of COVID-19 preventive measures comprised three domains with a total of 23 items. These domains were Mask usage (7 items; score range: 7-14), Handwashing (8 items; score range: 8-16), and social distancing (8 items; score range: 8-16). All 23 items had dichotomous answers. The adequacy of knowledge was determined based on the mean value of each domain. For mask usage, a score of more than 11 was considered adequate, while for handwashing and social distancing, scores of more than 13 were considered adequate. Knowledge of mask usage norms for children was deemed adequate if participants were aware of at least one guideline provided for mask usage in children up to 18 years old. The guidelines include a) No masks are required for children less than 5 years old; b) Mask usage for children aged 6-12 years should be done under supervision; c) Children aged 12 years and older should use masks like adults.

Behavioral surveillance aimed to understand and assess patterns and trends in people's behaviors and responses to the pandemic. The focus was on participants' practices regarding COVID-19 preventive measures, such as outdoor mask usage, the type of mask commonly used, frequency of usage, disposal methods, adherence to WHO handwashing steps, and adherence to social distancing norms in outdoor settings and gatherings. Handshaking avoidance was also evaluated.

The behavioral component of COVID-19 preventive measures consisted of three domains with a total of 23 items. These domains were mask usage (6 items; score range: 2-12), handwashing (10 items; score range: 10-20), and social distancing (7 items; score range: 7-14). Similar to the knowledge component, all 23 items had dichotomous answers, and the adequacy of behavior was determined based on the mean value of each domain. For mask usage, a score of more than 18 was considered adequate, while for handwashing and social distancing, scores of more than 15 and 11, respectively, were considered adequate.

Volunteer recruitment process

The data collection procedure involved the participation of CRMI students who were posted in the Rural Health Training Centre (RHTC) of the tertiary hospital. Before the commencement of the study, the students received a two-day training session on data collection. This training aimed to equip them with the necessary skills and knowledge required for effectively carrying out the data collection process.

Data collection procedure

According to the systematic random sampling method, the volunteers visited every 10th house. They explained the purpose of the study to the residents and obtained written consent from those willing to participate. The volunteers administered a pre-validated, semi-structured questionnaire in the local language to collect information on socio-demographic profiles, behavior related to COVID-19, and preventive measures. The interview lasts for about 20 mins. The information provided by the participants was treated as confidential, and their anonymity was maintained throughout the study. Participants who demonstrated poor knowledge and practices regarding COVID-19 preventive measures received health education at the conclusion of the study.

Statistical analysis

The collected data were entered into Microsoft Excel and analyzed using IBM Corp. Released 2011. IBM SPSS Statistics for Windows, Version 20.0. Armonk, NY: IBM Corp. [8], adhering to US English conventions. Descriptive statistics, including frequencies and percentages, were utilized to describe the behavioral practices related to COVID-19 and its preventive measures. The chi-square test was employed to assess the association between behavior and knowledge of COVID-19 preventive measures.

A linear regression analysis was conducted to examine the relationship between the overall knowledge score and the behavioral practice scores on mask usage, handwashing, and social distancing and to identify factors influencing knowledge and behavior. A p-value of less than 0.05 was considered statistically significant, indicating a significant association between the variables.

## Results

A total of 422 study participants participated in the survey. Approximately 63% of them belonged to the age group of ≤30 years. Nearly 46% of the participants were employed, and among them, 47% had frequent contact with COVID-19 (Table 1).

**Table 1:** Socio-demographic details of the study participants (n=422)

Characteristics		Frequency	Percentage
Age(years)	≤30	268	63.5
	>30	154	36.5
Gender	Male	198	47
	Female	224	53
Education	Postgraduate and above	86	20.3
	Graduate	196	46.4
	Secondary education	78	18.5
	Primary education	22	5.2
	Illiterate	40	9.1
Employment status	Employed	195	46.2
	Unemployed	227	53.8
Employment-frequent contact with covid (n=195)	Yes	92	47
	No	103	53
Socio-economic status as per Modified BG prasad Classification	Class 1	263	62.3
	Class 2	81	19.2
	Class 3	38	9.0
	Class 4	33	7.8
	Class 5	7	1.7

Out of the total 422 participants, 400 (94.7%) reported being aware of COVID-19, while 22 (5.3%) participants reported not being aware of it. Participants were asked about the common symptoms of COVID-19. The most frequently reported symptom was fever, identified by 205 participants (51.3%). Other commonly recognized symptoms included cough (113 participants, 28.3%), difficulty in breathing (86 participants, 21.5%), headache (45 participants, 11.3%), loose stool (20 participants, 5.0%), loss of taste and smell (20 participants, 5.0%), myalgia (muscle pain) (15 participants, 3.8%), and vomiting (12 participants, 3.0%) (Table 2).

**Table 2:** Frequency distribution of knowledge on COVID-19 and its preventive measures among the study participants (n=400) *Multiple response

Knowledge of COVID-19 and its preventive measures		Frequency	Percentage
Awareness of COVID-19 disease (n=422)	Yes	400	94.7
	No	22	5.3
Common symptoms of COVID-19*	Fever	205	51.3
	Cough	113	28.3
	Difficulty in Breathing	86	21.5
	Headache	45	11.3
	Loose stool	20	5.0
	Loss of taste and smell	20	5.0
	Myalgia	15	3.8
	Vomiting	12	3.0
Handwashing knowledge	Adequate	286	71.5
	Inadequate	114	28.5
Social distancing knowledge	Adequate	221	55.3
	Inadequate	179	44.7
Mask usage in preventing transmission of COVID-19	Adequate	248	62
	Inadequate	152	38
Reuse advisory for triple-layered masks by an infected patient	Yes	125	31.2
	No	275	68.8
Mask usage norms for children	Adequate	371	92.7
	Inadequate	29	7.3

Regarding handwashing, 286 (71.5%) reported having adequate knowledge, while 114 (28.5%) reported inadequate knowledge. A total of 221 participants (55.3%) reported having adequate knowledge of social distancing norms, and 248 (62%) reported adequate knowledge of mask usage in preventing the transmission of COVID-19. Regarding the reuse advisory for triple-layered masks by an infected patient, 125 (31.2%) were aware of it (Table 2).

In terms of mask usage behavior in outdoor settings, only 45 (11.3%) reported always using masks, while the majority, 355 (88.7%), reported partial usage. Among those who reported partial mask usage (355 participants), the most commonly mentioned places were crowded (255 participants, 71.8%), shopping areas (243 participants, 68.5%), hospital visits (132 participants, 37.2%), and temple visits (62 participants, 17.5%) (Table 3). 

**Table 3:** Frequency distribution of behavior surveillance on COVID-19 preventive measures among the study participants (n=400) *multiple response #Partially wearing masks refers to a situation where individuals do not fully comply with the recommended guidelines for wearing masks.

Characteristics		Frequency	Percentage
Mask usage in the outdoors	Yes, always	45	11.3
	Yes, partially	355	88.7
Places of partial^#^ usage of mask* (n=355)	Crowded places	255	71.8
	Shopping area	243	68.5
	Hospital visit	132	37.2
	Temple visit	62	17.5
The common type of mask used	Cloth mask	257	64.3
	Triple layer mask	135	33.7
	N95/ Carbon masks	8	2
Number of times single mask used	Once	85	21.3
	2-5 times	95	23.8
	More than 5 times	137	34.3
	Till it gets wet or dirt	83	20.8
The disposal method of face mask after usage*	Home Dustbin -open	243	60.8
	Closed bin	85	21.3
	On-street	26	6.5
	Street dustbin	21	5.3
	Cover with newspaper and put into the dustbin	16	4.0
	Burn	9	2.3
Separate dustbins for mask disposal	Yes	135	34.4
	No	257	65.6
Followed WHO-recommended steps for mask usage	Yes	52	13
	No	348	87
Hand washing after coming from outdoors	Yes	43	10.8
	No	357	89.2
Maintaining social distance at outdoors	Yes	87	21.7
	No	313	78.3

The majority of participants, 257 (64.3%), reported using cloth masks, while 135 participants (33.7%) reported using triple-layer masks. Only eight participants (2%) reported using other types of masks. The most common method of disposing of face masks after usage was in home dustbins (243 participants, 60.8%). Other reported methods included using closed bins (85 participants, 21.3%), on-street disposal (26 participants, 6.5%), street dustbins (21 participants, 5.3%), covering with newspaper and putting into the dustbin (16 participants, 4.0%), and burning (nine participants, 2.3%) (Table 3). 

Regarding the use of separate dustbins for mask disposal, 135 participants (34.4%) reported having separate dustbins, while 257 participants (65.6%) did not have separate dustbins. Out of the total participants, 43 (10.8%) reported washing their hands after coming from outdoors, while the majority, 357 participants (89.2%), reported not practicing handwashing. Additionally, 87 participants (21.7%) reported maintaining social distance outdoors (Table 3).

Table 4 provides insights into the participants' adherence to the WHO-recommended steps for face mask usage, highlighting the percentages of individuals who followed each step. Only 52 participants (13%) reported following the WHO-recommended steps for mask usage. 

**Table 4:** Frequency distribution on the practice of face mask usage by the WHO recommended steps (n=400)

WHO recommended steps for face mask usage	Number of participants followed n (%)
Before putting on a mask, clean hands with alcohol-based hand rub or soap and water	58 (14.5)
Cover mouth and nose with mask and make sure there are no gaps between your face and the mask	52 (13.0)
Avoid touching the mask while using it; if you do, clean your hands with alcohol-based hand rub or soap and water	80 (20.0)
Replace the mask with a new one as soon as it is damp and do not reuse single-use masks	56 (14.0)
To remove the mask; remove it from behind (do not touch the front of mask); discard immediately in a closed bin	115 (28.7)
Clean hands with alcohol-based hand rub or soap and water after removing	36 (09.0)
Followed all the steps recommended by WHO	52 (13.0)

Table 5 presents the association between behavior and knowledge of COVID-19 preventive measures. There was a statistically significant association between mask usage practice, type of mask used, separate bins for mask disposal, following WHO-recommended steps for mask usage, and knowledge (p-value < 0.05). There was no statistically significant association (p = 0.927) between knowledge of handwashing and handwashing behavior. However, there was a statistically significant association (p = 0.0006) between knowledge of social distancing and social distancing behavior. 

**Table 5:** Association between behavior surveillance and knowledge of COVID-19 preventive measures among the study participants *chi-square test; p-value <0.05, statistically significant

COVID-19 preventive measures		Adequate n (%)	Inadequate n (%)	p-value^#^
Mask usage behavior		Knowledge of mask usage		
Mask usage practice	Always	37 (82.2)	8 (17.8)	0.003*
	Partially	211 (59.4)	144 (40.6)	
Type of mask used	Cloth	131 (50.9)	126 (49.1)	<0.001*
	Triple layer and other masks	117 (81.8)	26 (18.2)	
Used separate bin at home	Yes	107 (79.3)	28 (20.7)	<0.001*
	No	141 (53.2)	124 (46.8)	
Followed WHO steps for mask usage	Yes	47 (90.4)	5 (9.6)	<0.001*
	No	201 (57.7)	147 (42.3)	
Number of times single mask used	Once	41 (48.2)	44 (51.8)	0.003
	More than once	207 (65.7)	108 (34.3)	
Proper disposal of face mask	Yes	53 (52.3)	48 (47.7)	0.02
	No	195 (65.7)	104 (37.8)	
Overall mask usage behavior		Knowledge of mask usage		
Mask usage	Adequate	47 (75.8)	15 (24.2)	0.014*
	Inadequate	201 (60.5)	137 (39.5)	
Handwashing behavior		Knowledge of handwashing		
Hand washing	Adequate	31 (72)	12 (28)	0.927
	Inadequate	255 (71.4)	102 (28.6)	
Social distancing behavior		Knowledge of social distancing		
Social distancing	Adequate	62 (71.2)	25 (28.8)	<0.001*
	Inadequate	159 (50.7)	154 (49.2)	

From Table 6, it is evident that age has a statistically significant positive relationship with all three domains of the dependent variable, which is knowledge of COVID-19 preventive measures. Similarly, gender also exhibits a statistically significant positive relationship. However, the variables of educational qualification, employment status, and socioeconomic status do not display statistically significant relationships with the dependent variable.

**Table 6:** A linear regression model predicting the influence of basic characteristics on the knowledge of COVID-19 preventive measures *p value <0.05, statistically significant

Basic characteristics	Knowledge on COVID-19 preventive measures									
	Mask usage			Handwashing			Social distancing			
	Standardized Coefficients Beta	t	p-value*	Standardized Coefficients Beta	t	p-value*	Standardized Coefficients Beta	t	p-value*	
(Constant)		3.734	.000		16.535	.000		13.589	.000	
Age	.405	8.967	.000	.322	6.839	.000	.322	6.741	.000	
Gender	.206	4.603	.000	.182	3.909	.000	.133	2.807	.005	
Education	.095	1.824	.069	.103	1.902	.058	.039	.710	.478	
Employment Status	-.047	-.976	.330	-.033	-.665	.506	-.017	-.330	.741	
Socio-economic status	.018	.364	.716	-.027	-.531	.596	-.012	-.236	.814	

From Table 7, it is observed that age has a statistically significant positive relationship with the domains of mask usage and social distancing. Similarly, gender also exhibits a statistically significant positive relationship, but only in the social distancing domain. However, the variables of educational qualification, employment status, and socioeconomic status do not demonstrate statistically significant relationships with the dependent variable. 

**Table 7:** A linear regression model predicting the influence of basic characteristics on the behavior of COVID-19 preventive measures p-value <0.05, statistically significant

Basic characteristics	Behavior on COVID-19 preventive measures								
	Mask usage			Handwashing			Social distancing		
	Standardized Coefficients Beta	t	p-value*	Standardized Coefficients Beta	t	p-value	Standardized Coefficients Beta	t	p-value*
(Constant)		10.341	.000		19.92	.000		10.92	.000
Age	.167	3.343	.001	-.049	-.964	.335	.225	4.598	.000
Gender	.062	1.254	.211	-.026	-.507	.612	.158	3.251	.001
Education	.028	.491	.623	.022	.380	.704	.072	1.277	.202
Employment Status	.035	.664	.507	-.007	-.138	.891	-.023	-.445	.656
Socio-economic status	-.024	-.430	.667	-.052	-.946	.345	-.012	-.218	.828

Table 8 presents the linear regression model for predicting the behavior of COVID-19 preventive measures based on knowledge. The standardized coefficient (Beta) for knowledge of mask usage is 0.152. This coefficient indicates that for every one standard deviation increases in knowledge on mask usage, the predicted value of behavior on mask usage by the study population increases by 0.152 standard deviations. There is a statistically significant positive relationship between knowledge and behavior on mask usage, indicating that the predicted value of behavior on mask usage increases with increased knowledge. This relationship is unlikely to be due to chance.

**Table 8:** A linear regression model predicting the influence of knowledge on the behavior of COVID-19 preventive measures p-value less than 0.05, statistically significant

Explanatory Variable		Behavioral Surveillance				
		Unstandardized coefficient		Standardized coefficient	t	p-value*
Mask usage	Constant	5.855	0.542		10.810	0.000
	Knowledge	0.142	0.046	0.152	3.065	0.002*
Handwashing	Constant	12.400	0.720		17.225	0.000
	Knowledge	0.043	0.051	0.042	0.834	0.405
Social distancing	Constant	5.709	0.603		9.468	0.000
	Knowledge	0.393	0.046	0.397	8.633	<0.001*

Similarly, the standardized coefficient (Beta) for knowledge of social distancing is 0.397. This coefficient suggests that for every one standard deviation increase in knowledge on social distancing, the predicted value of behavior on social distancing increases by 0.397 standard deviations. There is a statistically significant positive relationship between knowledge and behavior on social distancing. As knowledge of social distancing increases, the predicted value of behavior in social distancing also increases. This relationship is highly unlikely to be due to chance. However, in the case of handwashing behavior, knowledge does not play a significant role in predicting the behavior (p > 0.05). This means that there is no statistically significant relationship between knowledge and behavior regarding handwashing (Table 8).

## Discussion

The present study aimed to investigate behavioral surveillance patterns and adherence to COVID-19 preventive measures among the rural population in Tamil Nadu. A sample of 422 rural adults aged 18 years and older were surveyed. Among the participants, the majority (63%) were in the age group of 30 years or younger. Approximately 53% of the participants were female, and around 46% were employed, with 47% of the employed individuals having frequent contact with COVID-19. These findings are consistent with previous studies conducted in different regions of Tamil Nadu, Andhra Pradesh, and Maharashtra [7,9-11], which also reported predominantly female participation (ranging from 51% to 62%) and a high percentage of individuals aged less than 30 years (ranging from 58% to 72%). However, Gupta P. et al. [12] reported a higher proportion of male participants and an older age group (over 30 years). These sociodemographic characteristics are important to consider as they contribute to the diversity of the participant sample and may have implications for the study outcomes or behaviors related to COVID-19.

The importance of studying knowledge on COVID-19 and its preventive measures is to gain insights into the current understanding of the virus and develop evidence-based strategies to enhance public awareness, promote behavioral change, and ultimately contribute to the control and prevention of COVID-19. In the present study, the majority of participants (94.7%) reported being aware of COVID-19. Fever was the most commonly reported symptom, followed by cough, difficulty breathing, headache, and others. Similar findings, demonstrating knowledge levels of over 75% on COVID-19 and its symptoms, were reported in various studies conducted in India and China by Pannerselvam et al. (90%); Amalakanti et al. (94%); Amar Tak et al. (88%); Chaudhary et al. (80%); Gupta P. et al. (90%); Kulkarni et al. (77%); Kumar et al. (99%) and Zhong et al. (90%) [7,9-15]. In contrast, a study conducted among vulnerable groups in Africa reported lower knowledge of COVID-19 and its preventive measures [16]. This indicates a relatively high level of knowledge and awareness of COVID-19 worldwide among diverse populations.

In the current study, around three-fourths of the participants had adequate knowledge of handwashing practices; approximately more than 55% of them were aware of social distancing norms adopted in the community and knew of mask utilization in preventing the transmission of the disease. Our study findings regarding higher knowledge of handwashing practices, social distancing norms, and mask utilization are consistent with the findings reported by Amar Taksande et al. [10], which showed a knowledge level of 94% for handwashing practices and 88% for social distancing and mask utilization. Similar findings were also reported in studies conducted by Zhong BL et al. and Kulkarni S et al., which also demonstrated higher knowledge levels of COVID-19 preventive measures [13-14]. These consistent findings across studies indicate a significant understanding and awareness of the importance of these preventive measures in controlling the spread of COVID-19. These consistent findings across studies suggest a widespread understanding and awareness of the importance of these preventive measures in controlling the spread of COVID-19. These findings suggest a relatively good level of knowledge regarding hand hygiene practices, which is a crucial preventive measure for reducing the spread of COVID-19. However, the study indicates that there is room for improvement in terms of maintaining an appropriate physical distance to prevent the spread of the virus and ensuring proper mask usage as preventive measures against COVID-19.

The behavioral surveillance of the study participants in the current study revealed that only 11% always wear masks when going outdoors, while the majority (88.7%) wear them partially. Among the partial mask users, 33.7% reported using a triple-layered mask, and 71.8% wore masks in crowded places. Only 13% of the participants followed all the steps recommended by the WHO. Consistent with our findings, a study conducted by Panneerselvam et al. in Tamil Nadu [7] and Kulkarni et al. in Karnataka [14] reported reduced adherence to mask usage (ranges 11% to 23%) while going outdoors and the proper method (WHO) of wearing a face mask. In contrast to our findings, various studies conducted in India and China reported higher compliance with mask usage (ranging from 60%-75%) outdoors and following the appropriate steps recommended by the WHO (45 to 70%) [9-13,15]. These variations could be attributed to varying levels of awareness programs and public health campaigns regarding the disease and its prevention in different geographical areas.

In this study, a statistically significant association was seen between behavioral surveillance on mask usage and knowledge on the same, such as adequacy of mask usage, type of mask used, the number of times the same mask was used, use of separate bins for disposal, following WHO steps in mask usage, and social distancing. However, no statistically significant association (p = 0.927) was found between knowledge of handwashing and handwashing behavior. Both participants with adequate and inadequate knowledge of handwashing exhibited similar rates of handwashing. A study from China by Zhong et al. [14] reported that the study participants had good knowledge, optimistic attitudes, and appropriate practices towards COVID-19 during the rapid rise period of the COVID-19 outbreak, and good knowledge was associated with optimistic attitudes and appropriate practices towards COVID-19. These findings suggest that health education programs aimed at improving COVID-19 knowledge help encourage an optimistic attitude and maintain safe practices among the target population.

The findings from the regression model in the present study suggest that age and gender have a significant impact on individuals' knowledge of COVID-19 preventive measures. The results indicate that as individuals grow older, their likelihood of having a greater understanding of these measures increases. Additionally, being female is associated with a higher level of knowledge. However, when it comes to actually adopting the behaviors related to COVID-19 prevention, age emerges as the main influencing factor, while variables such as occupation, socio-economic status, and education do not show a significant association. This finding is consistent with a study conducted by Zhong BL et al. [14], which also found that age, gender, and education play vital roles in influencing knowledge and the adoption of COVID-19 preventive behaviors. These findings suggest that older individuals are more likely to engage in the recommended preventive behaviors compared to younger individuals.

Upon analyzing linear regression to estimate the impact of knowledge on behavior, we observed that knowledge significantly influences mask usage and social distancing behaviors but not handwashing behavior. These findings suggest that improving knowledge about COVID-19 preventive measures can have a positive effect on specific aspects of behavioral surveillance, particularly in relation to mask usage and social distancing. However, it is noteworthy that the study did not find a significant impact of handwashing knowledge on actual handwashing behavior (p>0.05). This implies that there may be a gap between knowledge and behavior when it comes to handwashing practices. It highlights the need to further analyze and address this gap to promote and encourage proper handwashing behavior among individuals. These findings emphasize the importance of targeted interventions and educational campaigns that focus not only on increasing knowledge about preventive measures but also on addressing barriers and promoting behavior change. Strategies should be tailored to specific behaviors and take into account factors that influence behavior, such as individual beliefs, social norms, and environmental factors. By addressing the gaps in behavior change, we can enhance the effectiveness of public health interventions and mitigate the spread of COVID-19.

Although this study provides valuable insights on the influence of knowledge on adopting COVID-19-appropriate behavior, it is important to acknowledge certain limitations. The reliance on self-reporting and recall biases could have influenced participant responses, potentially affecting the accuracy and reliability of the data. Additionally, the study focused on a limited set of variables in assessing the external factors influencing knowledge and behavior. To address these limitations and further advance our understanding, future research should consider larger and more diverse samples. By including participants from different demographic backgrounds and geographic locations, we can enhance the generalizability of the findings. Longitudinal designs could also be employed to capture changes in knowledge and behavior over time and identify potential causal relationships.

Furthermore, it would be beneficial to incorporate objective measures of COVID-19 preventive measures, such as direct observations or measurements, to supplement self-reported data. This would provide a more comprehensive and reliable assessment of individuals' actual practices. By addressing these considerations, future studies can provide a more robust and comprehensive understanding of the factors influencing knowledge and behavior related to COVID-19 preventive measures, ultimately contributing to the development of effective interventions and strategies to combat the pandemic.

## Conclusions

In the present study, it was found that the majority of participants had adequate knowledge about COVID-19. However, when assessing their actual behavioral practices, it was observed that there was a lack of adherence to proper hand hygiene, social distancing, and wearing masks according to WHO guidelines. The study revealed a significant association between knowledge and behavior. Specifically, knowledge significantly influenced mask usage and social distancing behaviors but not handwashing behavior. These findings emphasize the crucial role of knowledge in promoting preventive practices for COVID-19. It is essential to enhance public awareness and education regarding different aspects of mask usage, including types of masks, proper usage, and safe disposal practices. By providing comprehensive information and guidance, it is possible to foster a wider and more effective adherence to mask guidelines, thereby helping to mitigate the spread of COVID-19.

The study also highlights the need to improve adherence to COVID-19 preventive measures, particularly among the rural population in Tamil Nadu. Targeted interventions and educational campaigns are necessary to promote and reinforce appropriate preventive behaviors, with specific attention to mask usage and hand hygiene. These measures can help bridge the knowledge-practice gap and contribute to the effective control of COVID-19 transmission in rural communities. Further research and interventions in this area are warranted to inform public health policies and promote safe behaviors during pandemics. By addressing the knowledge-practice gap and implementing targeted strategies, it is possible to enhance the overall response to COVID-19 and protect the health and well-being of individuals in rural areas and beyond.
